# The role of surfactant protein D in the colonisation of the respiratory tract and onset of bacteraemia during pneumococcal pneumonia

**DOI:** 10.1186/1465-9921-6-126

**Published:** 2005-10-28

**Authors:** R Jounblat, H Clark, P Eggleton, S Hawgood, PW Andrew, A Kadioglu

**Affiliations:** 1 Department of Infection Immunity and Inflammation, University of Leicester, Leicester, LE1 9HN, UK; 2 MRC Immunochemistry Unit, University of Oxford, South Parks Road, Oxford, OX1 3QU, UK; 3 Institute of Biomedical and Clinical Sciences, Peninsula Medical School, Exeter, EX1 2LU, UK; 4 Cardiovascular Research Institute and Department of Paediatrics, University of California, San Francisco, San Francisco, California, USA

**Keywords:** *Streptococcus pneumoniae*, surfactant protein D, respiratory tract

## Abstract

We have shown previously that surfactant protein D (SP-D) binds and agglutinates *Streptococcus pneumoniae in vitro*. In this study, the role of SP-D in innate immunity against *S. pneumoniae *was investigated *in vivo*, by comparing the outcome of intranasal infection in surfactant protein D deficient (SP-D-/-) to wildtype mice (SP-D+/+). Deficiency of SP-D was associated with enhanced colonisation and infection of the upper and lower respiratory tract and earlier onset and longer persistence of bacteraemia. Recruitment of neutrophils to inflammatory sites in the lung was similar in both strains mice in the first 24 hrs post-infection, but different by 48 hrs. T cell influx was greatly enhanced in SP-D-/- mice as compared to SP-D+/+ mice. Our data provides evidence that SP-D has a significant role to play in the clearance of pneumococci during the early stages of infection in both pulmonary sites and blood.

## Introduction

*Streptococcus pneumoniae *is a major human pathogen responsible for respiratory tract infections, septicaemia and meningitis. The pneumococcus is particularly well adapted to colonising the mucosal surfaces of the nasopharynx and the combination of bacterial virulence factors and the manipulation of host tissue components allow the pneumococcus to spread from the nasopharynx to sterile regions of the lower respiratory tract, leading to infections such as pneumonia. In the early stages after infection, natural pulmonary defence mechanisms are required for efficient clearance of the pneumococcus. Recent studies have drawn attention to the important role of lung surfactant protein D (SP-D) as the first line of defence in natural innate immunity to microbial invasion of the respiratory tract, involved in the binding, aggregation, and phagocytic uptake of invading micro-organisms [1-4]. In addition, SP-D has also been shown to be involved in binding to apoptotic polymorphonuclear leukocytes and alveolar macrophages to enhance their clearance by healthy resident macrophages [5].

SP-D, is a member of the collectin family that also includes mannose binding lectin (MBL), conglutinin, collectin-43 and surfactant protein A (SP-A). It is predominantly found in the respiratory tract, but is also detected at other non-pulmonary mucosal surfaces such as the salivary and lachrymal gland, ovary, uterus, oesophagus, stomach, testes, thyroid, heart and kidney [4,6,7]. In the lung, SP-D is secreted by alveolar type II cells and by non-ciliated Clara cells as dodecamers consisting of four collagenous trimers cross-linked by disulphide bonds, to create a cruciform structure. Each trimer of the molecule consists of three polypeptide chains and each subunit consists of four domains: a short amino acid terminal end, a collagen-like region followed by a short α-helical region and a C-type carbohydrate recognition domain (CRD) responsible for its lectin activity [1,2,8,9].

A number of pulmonary pathogens, including *Streptococcus pneumoniae*, have been reported to be agglutinated by lung surfactant protein D *in vitro *[10-13]. In one such study using SP-D knockout mice (SP-D-/-), the *in vivo *requirement for SP-D in the early pulmonary clearance and modulation of the inflammatory response to bacterial pathogens was shown. Although increased inflammation, oxidant production and decreased macrophage phagocytosis were associated with SP-D deficiency in the lungs of mice, killing of Gram-negative (*Haemophilus influenzae*) and Gram-positive (group B streptococcus) bacteria was unaltered [14]. In another study, a decrease in viral clearance and an increase in production of inflammatory cytokines were detected in response to viral challenge in SP-D-deficient mice when compared to control mice [15]. Furthermore, treatment of wild-type mice with native full length SP-D or recombinant SP-D substantially increased their survival rate in mice challenged intranasally with *Aspergillus fumigatus *spores [16] and recombinant SP-D promoted the clearance of fungal spores from the mouse lung (Howard Clark et al., unpublished).

Another study reported that highly multimerised SP-D molecules bound to strains of serotype 4, 19 and 23 *S. pneumoniae*, causing their agglutination and enhancing their uptake by neutrophils [17]. More recently, we showed that recombinant human SP-D, expressed in *Escherichia coli*, consisting of the head and neck regions of the native molecule, bound to all strains of *S. pneumoniae *that were tested, but the extent of binding varied between strains. Full-length native SP-D aggregated pneumococci in a calcium-dependent manner *in vitro*, but the aggregation of pneumococci varied not only between strains of the same multilocus sequence type (but different serotypes), but also between strains of the same serotype. Neither recombinant truncated SP-D nor native full-length SP-D enhanced killing of pneumococci by human neutrophils in the absence of serum however [11].

Given the above findings, we hypothesise that SP-D has an important role to play in the innate immune defence of the upper and lower respiratory tract against pneumococcal infection *in vivo*, by promoting the agglutination and subsequent clearance of *S. pneumoniae*. This would prevent the colonisation of the nasopharynx and subsequently limit the spread of pneumococci from the upper to the lower respiratory tract by enhancing clearance via the mucocilliary system, thus allowing enough time for other components of both the innate and adaptive immune system to come into play. In the present study we assessed the *in vivo *contribution of SP-D to host defence by intranasally infecting SP-D-deficient and sufficient mice with *S. pneumoniae*. Bacterial growth kinetics in the nasopharynx, trachea, lungs and blood, development of lung pathology and host inflammatory leukocyte infiltration into lungs was compared in both strains of mice following infection.

## Methods

### Source of mice

Wild-type control C57BL/6 mice were obtained from Harlan Olac (Bicester, UK) and SP-D genes were ablated by gene targeting of embryonic stem cells, backcrossed 10 generations into the C57BL/6 genetic background, and maintained at the animal house of the Department of Biochemistry, Oxford University under barrier facilities [5,18]. All mice were at least 8 weeks old at use and did not have detectable levels of anti-type 2 antibodies. All experimental protocols were approved by appropriate U.K. Home Office licensing authorities and by the University of Leicester Ethical Committee.

### Bacteria

*Streptococcus pneumoniae *serotype 2, strain D39 was obtained from the National Collection of Type Cultures, London, UK (NCTC 7466). Bacteria were identified as pneumococci prior to experiments by Gram stain, catalase test, α-haemolysis on blood agar plates and by optochin sensitivity. To obtain virulent pneumococci, bacteria were cultured and passaged through mice as described previously [19] and subsequently recovered and stored at -80°C. When required, suspensions were thawed at room temperature and bacteria harvested by centrifugation before re-suspension in sterile phosphate buffered saline (PBS).

### Intranasal challenge of mice with *S. pneumoniae*

As previously described, [19] mice were infected intranasally with 1 × 10^6 ^CFU *S. pneumoniae*. At pre-chosen intervals following infection, groups of mice were deeply anaesthetised with 5% (v/v) fluothane (Astra-Zeneca, Macclesfield, UK) and blood was collected by cardiac puncture. Mice were killed by cervical dislocation, and the lungs, trachea and nasopharynx were removed separately into 10 ml of sterile PBS, weighed and then homogenised in a hand held homogeniser (Fischer Scientific, UK). Viable counts in homogenates and blood were determined by serial dilution in sterile PBS and plating onto blood agar plates as previously described [19].

### Pathology

At intervals following infection, lungs were excised, embedded in Tissue-Tec OCT (Sakura), and frozen in liquid nitrogen with an isopentane heat buffer to prevent snap freezing and tissue damage. Samples were stored at -80°C. Sections (10 μm) were taken at -18°C on a Bright cryostat and then allowed to dry at room temperature. Sections from throughout the lung were taken with at least thirty sections per lung being analysed. Following acetone fixation, the sections were stained with haematoxylin and eosin and fixed with DPX mountant (BDH) for permanent storage [19]. Lung pathology was scored blind on the following criteria; cellular infiltration around bronchioles, perivascular and peribronchial areas, hypertrophy of bronchiole walls, and oedema.

### Immunohistochemistry

As described previously [19], leukocyte recruitment into lung tissue was analysed by an alkaline phosphatase anti-alkaline phosphatase (APAAP) antibody staining method. Rat anti-mouse monoclonal antibodies to T cells (anti-CD3), B cells (anti-CD19), macrophages (anti-F4/80) and neutrophils (anti-Gr-1) (Serotec, Oxford, UK) and secondary rabbit anti-rat antibody (Dako, Denmark) and rat APAAP antibody were used as previously described on infected lung tissue sections. Sections from throughout the lung were taken with at least twenty sections per lung being analysed. Tissue sections (approximately twenty sections from each lung at chosen time points) were used for each antibody to be tested, along with 3 sections for negative controls which consisted of using an isotype matched control antibody; excluding the primary antibody (or the secondary enzyme conjugated antibody); or not incubating with the substrate-chromogen solution. Finally, the sections were washed and counterstained briefly with haematoxylin and mounted in aqueous mounting medium (Aquamount, DAKO). Once stained, each section was quantified double blind by two observers (RJ and AK). Positively stained cells within the vicinity of inflamed bronchioles were enumerated within the 1 mm^2 ^area of a counting grid. Twenty individual grids per each tissue section were quantified, making a total of 400 grids counted per lung per each time point (20 tissue sections in total per each antibody tested). A total of four lungs per time point were analysed.

### Statistical analysis

Comparisons of bacterial loads between mouse strains or treatments were made with unpaired Students *t *tests. Statistical significance was considered at P values <0.05.

## Results

### The role of SP-D in upper and lower respiratory tract pneumococcal colonisation Nasopharynx

**Figure 1:**
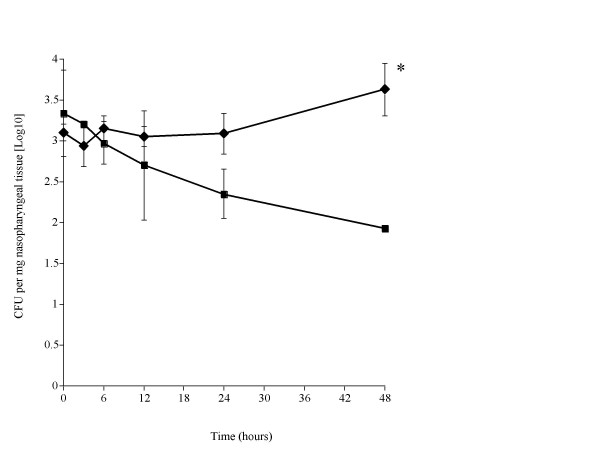
Time course of the change in numbers of *S. pneumoniae *in the nasopharynx (figure 1), trachea (figure 2), lungs (figure 3) and blood (figure 4) of SP-D-/- (◆) and SP-D+/+ (■) mice infected intranasally with 10^6 ^CFU (n = 10 mice at each time point, error bars indicate SEM). * denotes P < 0.01, ** denotes P < 0.05 for SP-D-/- when compared to wildtype at equivalent time point.

*S. pneumoniae *successfully colonised the nasopharynx of SP-D-/- mice, but were cleared from SP-D+/+ mice. Pneumococcal numbers in the nasopharynx of SP-D-/- mice remained unchanged over the 48 hr period post infection (Fig. 1) whereas pneumococcal numbers were significantly reduced in SP-D+/+ mice by 48 hrs post infection as compared to SP-D-/- mice (P < 0.01). SP-D+/+ mice eventually cleared the pneumococci in their nasopharynx by 72 hrs (by which time-point the experiment was ended) while SP-D-/- mice remained colonised at the same rate at this time-point (data not shown).

### Trachea

**Figure 2:**
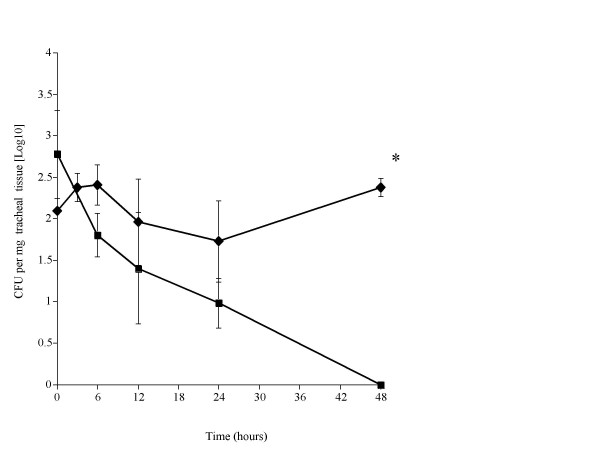
Time course of the change in numbers of *S. pneumoniae *in the nasopharynx (figure 1), trachea (figure 2), lungs (figure 3) and blood (figure 4) of SP-D-/- (◆) and SP-D+/+ (■) mice infected intranasally with 10^6 ^CFU (n = 10 mice at each time point, error bars indicate SEM). * denotes P < 0.01, ** denotes P < 0.05 for SP-D-/- when compared to wildtype at equivalent time point.

Differences between SP-D+/+ and SP-D-/- mice were also apparent in the colonisation of the trachea by pneumococci. Numbers of pneumococci remained constant over the 48 hr period post-infection period in SP-D-/- mice, whereas bacteria were cleared from the trachea of SP-D+/+ mice by 48 hrs post-infection (P < 0.01, compared to SP-D-/- mice) (Fig. 2).

### Lungs

**Figure 3:**
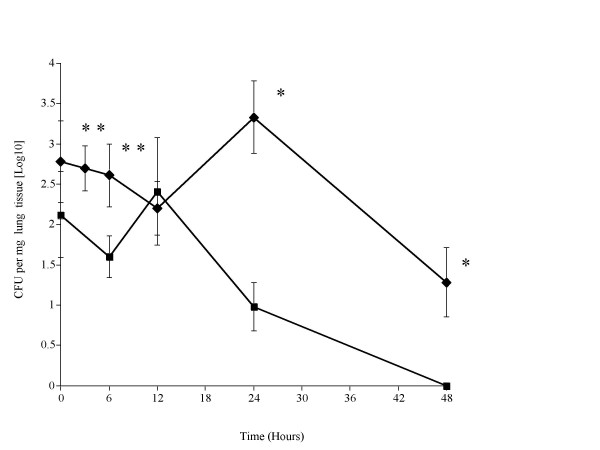
Time course of the change in numbers of *S. pneumoniae *in the nasopharynx (figure 1), trachea (figure 2), lungs (figure 3) and blood (figure 4) of SP-D-/- (◆) and SP-D+/+ (■) mice infected intranasally with 10^6 ^CFU (n = 10 mice at each time point, error bars indicate SEM). * denotes P < 0.01, ** denotes P < 0.05 for SP-D-/- when compared to wildtype at equivalent time point.

Pneumococcal growth in the lungs of SP-D-/- mice was significantly greater at 6, 24 and 48 hrs (P < 0.05) post-infection when compared to SP-D+/+ mice (Fig. 3). The number of pneumococci in SP-D-/- lungs increased over the 24 hr period post infection, whereas numbers of pneumococci in the lungs of SP-D+/+ mice decreased over this same period (P < 0.05 compared to time zero). Thereafter, numbers of pneumococci recovered from lungs declined significantly (P < 0.05 compared to 24 hrs) in both mice by 48 hrs post-infection (Fig. 3). Pneumococci were cleared from SP-D+/+ mice by 48 hrs post-infection and by 54 hrs for SP-D-/- mice (data not shown for this time-point).

### The role of SP-D in bacteraemia

**Figure 4:**
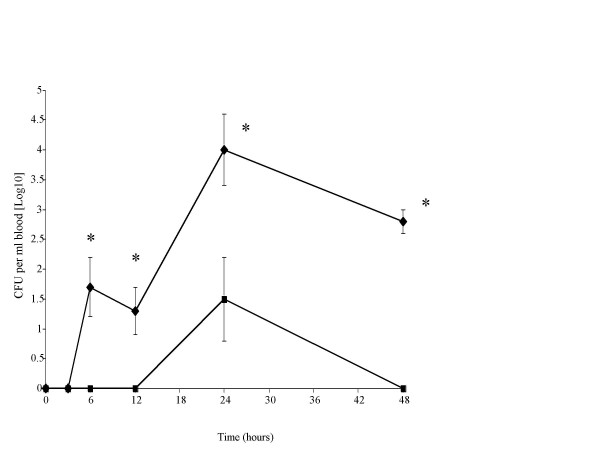
Time course of the change in numbers of *S. pneumoniae *in the nasopharynx (figure 1), trachea (figure 2), lungs (figure 3) and blood (figure 4) of SP-D-/- (◆) and SP-D+/+ (■) mice infected intranasally with 10^6 ^CFU (n = 10 mice at each time point, error bars indicate SEM). * denotes P < 0.01, ** denotes P < 0.05 for SP-D-/- when compared to wildtype at equivalent time point.

In the blood of SP-D-/- mice (Fig. 4), pneumococci were recovered as early as 6 hrs after infection and bacterial numbers were further increased by 24 hrs (P < 0.05, compared to 6 and 12 hrs). In contrast, pneumococci were not detected in blood of SP-D+/+ mice at 6 and 12 hrs post infection and by 24 hrs was present in significantly lower numbers (P < 0.01) as compared to SP-D-/- mice at equivalent time-point. These bacteria were eventually cleared in SP-D+/+ mice by 48 hr post-infection whereas they were still present in the blood of SP-D-/- mice by 48 hrs, albeit at a lower level (Fig. 4).

### Development of pathology in SP-D-/- and SP-D+/+ lungs infected with *S. pneumoniae*

**Figure 5:**
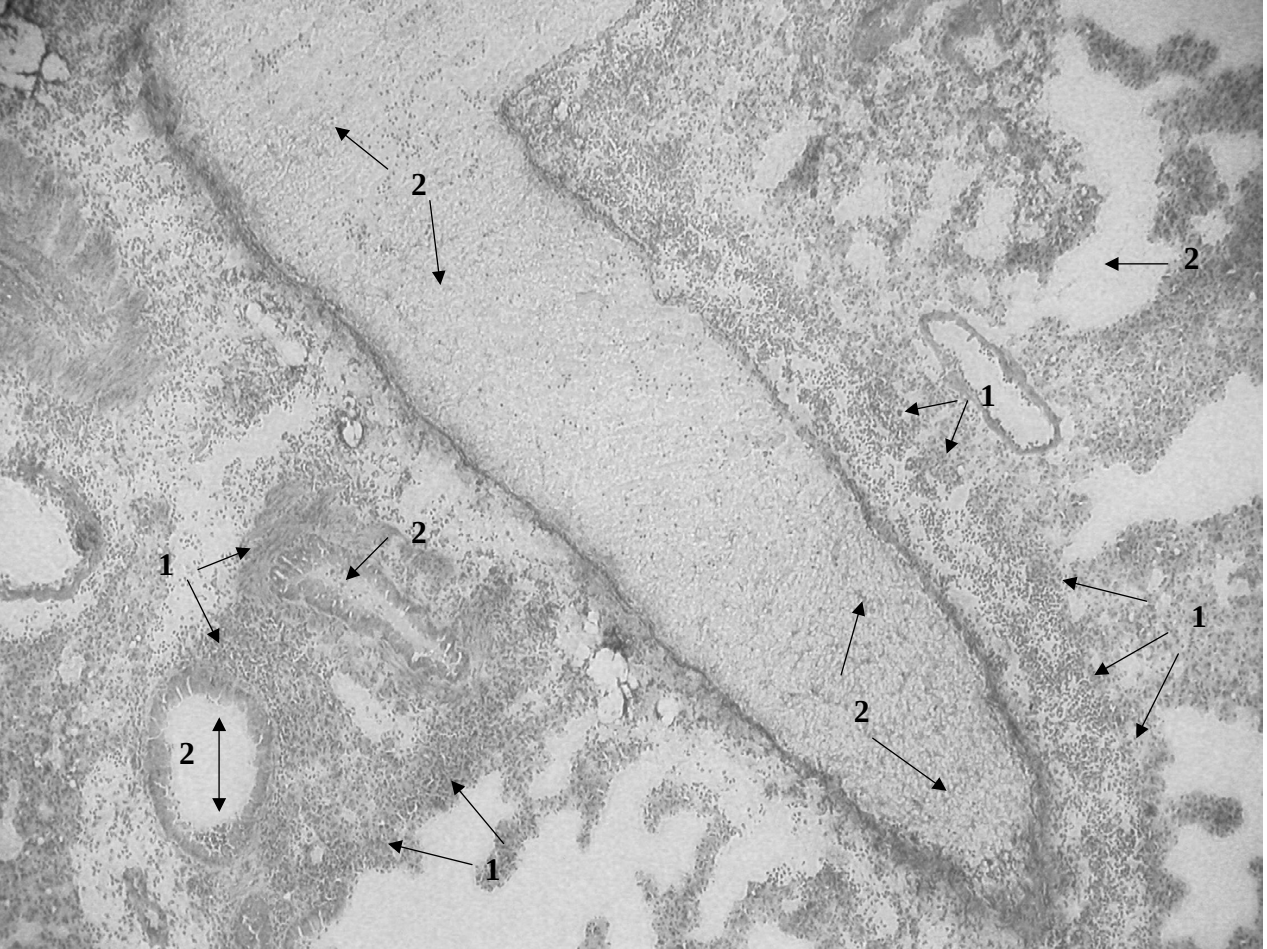
Light microscopy of lung tissue from mice infected with 10^6^CFU of *S. pneumoniae*. SP-D-/- 24 h post-infection (figure 5), SP-D+/+ 24 h post-infection (figure 6), SP-D-/- 48 h post-infection (figure 7) and SP-D+/+ 48 h post-infection (figure 8). Magnification ×250 for figures 5 and 8, ×400 for figures 6 and 7. See results for description of arrows.

**Figure 6:**
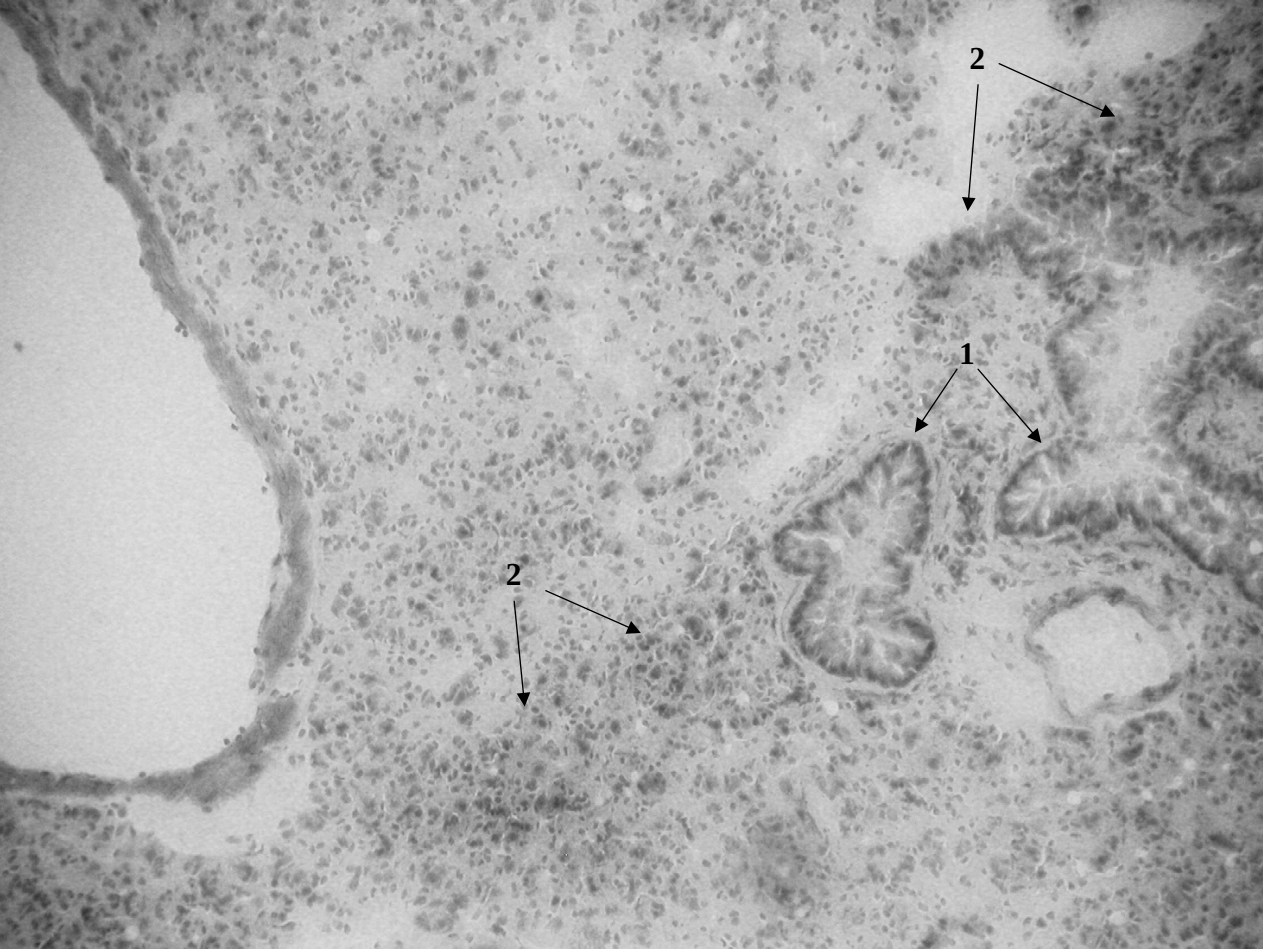
Light microscopy of lung tissue from mice infected with 10^6^CFU of *S. pneumoniae*. SP-D-/- 24 h post-infection (figure 5), SP-D+/+ 24 h post-infection (figure 6), SP-D-/- 48 h post-infection (figure 7) and SP-D+/+ 48 h post-infection (figure 8). Magnification ×250 for figures 5 and 8, ×400 for figures 6 and 7. See results for description of arrows.

**Figure 7:**
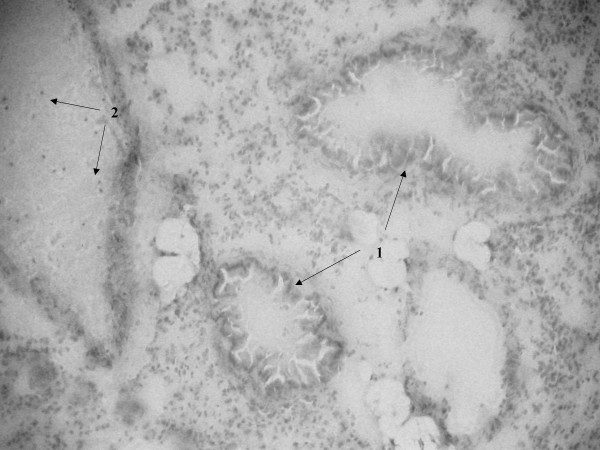
Light microscopy of lung tissue from mice infected with 10^6^CFU of *S. pneumoniae*. SP-D-/- 24 h post-infection (figure 5), SP-D+/+ 24 h post-infection (figure 6), SP-D-/- 48 h post-infection (figure 7) and SP-D+/+ 48 h post-infection (figure 8). Magnification ×250 for figures 5 and 8, ×400 for figures 6 and 7. See results for description of arrows.

**Figure 8:**
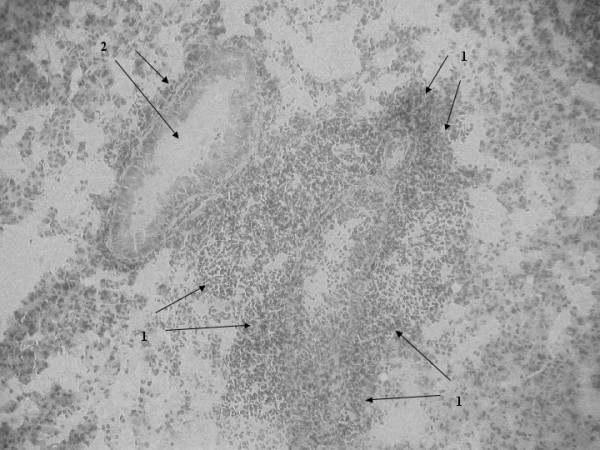
Light microscopy of lung tissue from mice infected with 10^6^CFU of *S. pneumoniae*. SP-D-/- 24 h post-infection (figure 5), SP-D+/+ 24 h post-infection (figure 6), SP-D-/- 48 h post-infection (figure 7) and SP-D+/+ 48 h post-infection (figure 8). Magnification ×250 for figures 5 and 8, ×400 for figures 6 and 7. See results for description of arrows.

Histopathological examination of lung tissue of SP-D+/+ and SP-D-/- mice infected with *S. pneumoniae *was done at time zero and at 24 and 48 hrs post infection. We have previously described in detail, the lung histology in non-infected SP-D-/- mice [5,18]. The histology of SP-D-/- mice used in the infection studies at time zero was the same as non-infected SP-D-/- mice. Briefly, histological changes in both non-infected SP-D-/- and infected SP-D-/- mice at time zero included increases in the size of alveolar type-II cells and scattered accumulation of material in the alveolar lumen (although many alveoli still remained unaffected), a marked increase in alveolar macrophage size, with many macrophages exhibiting a foamy appearance. Other than these well-documented features however, these mice exhibited no further histological evidence of lung inflammation or injury, consistent with the apparent health of these mice (data not shown as we have extensively described these features before) [5,18]. By 24 hrs post-infection however, SP-D-/- lungs exhibited features that were not apparent at time zero. These included heavy cellular infiltration visible around infected bronchioles and perivascular areas (Fig. 5, arrows-1) and increased inflammation characterised by exudate and thickening of the bronchiolar walls secondary to inflammation (Fig. 5, arrows-2). The same extent of bronchiole wall thickening was seen in the lungs of both SP-D-/- and SP-D+/+ mice at 24 hrs post infection but there was considerably less cellular infiltration into peribronchial and perivascular areas of SP-D+/+ lungs when compared to SP-D-/- lungs (Fig. 6, arrows 1 for bronchiole wall thickening, arrows 2 for cellular infiltration). However, by 48 hrs post-infection, peribronchial and perivascular cellular infiltration into SP-D-/- lungs had decreased significantly (Fig. 7, arrows 1 & 2) but had increased in SP-D+/+ lungs as compared to SP-D-/- mice (Fig. 8, arrows 1 for cellular infiltration & arrows 2 for bronchial inflammation).

### Analysis of leukocyte infiltration into lungs of SP-D+/+ and SP-D-/- mice following pneumococcal infection

After intranasal challenge, leukocyte infiltration patterns into SP-D-/- and SP-D+/+ lungs were analysed at time zero, 24 and 48 hrs post-infection (Table-1A). In both strains, increased recruitment of neutrophils into inflamed areas of lung tissue was detected within the bronchiolar lumen, in the bronchiole wall and also in the perivascular areas within the vicinity of inflamed bronchioles. At 24 hrs post-infection, in areas of inflamed bronchioles of both mouse strains, numbers of neutrophils increased in significant numbers (P < 0.01, compared to time zero values for both strains, Table-1A). There was no significant difference between the strains at this time-point when compared to each other. However, by 48 hrs post-infection, there was a significant decrease in SP-D-/- mouse lung neutrophil numbers (P < 0.05 compared to 24 hrs) whereas the number of neutrophils in SP-D+/+ lungs at 48 hrs further increased as compared to 24 hrs and as compared to SP-D-/- mice at equivalent timepoint (P < 0.05, Table-1A). Overall, there was a 7.7 fold increase in neutrophil numbers by 24 hrs in SP-D-/- mice as compared to time zero, which dropped to a 5.2 fold increase by 48 hrs post-infection. In SP-D+/+ mice, there was a smaller 5.4 fold increase in neutrophil numbers by 24 hrs as compared to time zero, however the proportion of neutrophils in these mice at 24 hrs (70% of total leukocyte population) was greater than that of SP-D-/- mice at equivalent timepoint (53% of total leukocyte population) and also so by 48 hrs post-infection (73% to 59%, SP-D+/+ to SP-D-/- respectively). Importantly, in contrast to SP-D-/- mice, the neutrophil influx in SP-D+/+ mice continued to increase by 6.5 fold by 48 hrs post-infection compared to time zero. In SP-D-/- mice the neutrophils influx had declined by this time point.

**Table 1:** Lung leukocyte populations in SP-D-/- and SP-D+/+ mice at time zero, 24 and 48 hrs post-intranasal pneumococcal challenge. Leukocyte subpopulations (neutrophils, T cells, macrophages and B cells) numerated in the vicinity of inflamed bronchioles were expressed as cells per mm^2 ^lung tissue. Leukocyte subpopulations expressed as the percentage of total lung leukocytes are shown in parenthesis. Fold increases in leukocytes subpopulations compared to time zero levels. N = 4 mice per each time point analysed for all samples. "a" denotes P < 0.01, "b" denotes P < 0.05 as compared to time zero values. "c" denotes P < 0.01, "d" denotes P < 0.05, SP-D-/- mice compared to SP-D+/+ mice at equivalent time-point.

***A) Neutrophils***	SP-D+/+ mice		SP-D-/- mice	
	
Time	Cells/mm^2^	Fold increase	Cells/mm^2^	Fold increase
	
Zero:	11 +/- 2		9 +/-2	
24:	60 +/-5 ^a ^(70%)	5.4	70+/-8^a ^(53%)	7.7
48:	72 +/-7 ^a ^(73%)	6.5	47 +/-4 ^b, d ^(59%)	5.2
				
***B) T cells***	SP-D+/+ mice		SP-D-/- mice	
	
Time	Cells/mm^2^	Fold increase	Cells/mm^2^	Fold increase
	
Zero:	9 +/-1		8 +/-2	
24:	9 +/-1 (11%)	no change	48 +/-8 ^a, c ^(36%)	6
48:	8 +/-1 (8%)	no change	16 +/-4 ^b, d ^(20%)	2
				
***C) Macrophage***	SP-D+/+ mice		SP-D-/- mice	
	
Time	Cells/mm^2^	Fold increase	Cells/mm^2^	Fold increase
	
Zero:	7 +/-1	no change	7 +/-1	
24:	8 +/-2 (9%)	no change	8 +/-3 (6%)	no change
48:	9 +/-2 (9%)	no change	8 +/-2 (10%)	no change
				
***D) B cells***	SP-D+/+ mice		SP-D-/- mice	
	
Time	Cells/mm^2^	Fold increase	Cells/mm^2^	Fold increase
	
Zero:	8 +/-1		6 +/-1	
24:	8 +/-2 (9%)	no change	7 +/-3 (5%)	no change
48:	9 +/-2 (9%)	no change	9 +/-1 (11%)	no change

There were, dramatic differences in lung tissue T cell accumulation between SP-D-/- and SP-D+/+ mice. In SP-D-/- lungs, T cell numbers showed a sharp 6-fold increase around inflamed bronchioles by 24 hrs post-infection (P < 0.01, when compared to time zero, see table-1B). T cell numbers then decreased to a 2-fold increase by 48 hrs post-infection (P < 0.05 as compared to time zero, see table-1B). In contrast, in the lungs of SP-D+/+ mice, there was no increase in the numbers of T cells in inflamed areas throughout the 48 hr post-infection period (P > 0.05, when compared to time zero). T cell numbers in SP-D+/+ were significantly lower than in SP-D-/- lungs at 24 and 48 hr post-infection (P < 0.01 for 24 hr and P < 0.05 for 48 hrs).

Macrophage and B cells numbers remained unchanged in the lungs of both SP-D-/- or SP-D+/+ (P > 0.05 as compared to time zero) over the 48 hr post-infection period (Table-1C &1D). PBS alone challenged mice had minimal leukocyte numbers in lungs, with each leukocyte population counted below 10 cells/mm^2 ^(data not shown).

## Discussion

Previous evidence has shown that SP-D interacts with *S. pneumoniae in vitro *[11,17]. The results of the current study are the first to demonstrate *in vivo*, that SP-D has an important role to play in pneumococcal clearance. Pneumococcal colonisation of the upper and lower respiratory tract, and infiltration patterns of leukocytes into the lungs of infected mice were affected by the absence of SP-D. Pulmonary clearance of intranasally administered *S. pneumoniae *was significantly reduced in SP-D deficient mice as compared to SP-D sufficient controls. Furthermore, our results clearly demonstrate that lack of SP-D allows persistent pneumococcal colonisation of the nasopharynx and trachea and early onset and increased levels of bacteraemia in colonised mice. Our results also indicate that SP-D influences the accumulation of T cells within the vicinity of inflamed bronchioles, whereby increased levels of T cell infiltration into SP-D deficient lungs was observed. This is the first report to demonstrate *in vivo*, that SP-D deficiency leads to increased pneumococcal colonisation of the nasopharynx and trachea, hastens the onset and development of bacteraemia, and affects leukocyte infiltration patterns into infected lungs.

SP-D is synthesised and secreted not only by pulmonary epithelial cells but also by epithelial cells and submucosal glands of the trachea of the normal adult mouse [20] and has been detected at low concentration (56 ng/ml) in nasopharyngeal washings of normal mice [21]. Based on our results in the nasopharynx and trachea it is clear that SP-D has a crucial role to play in these sites during pneumococcal infection. Consequently, it is clear therefore that SP-D prevents persistent upper airway colonisation by pneumococci and helps protect against invasion of the lower airways. However, it is also conceivable that lack of SP-D may affect resident leukocyte populations involved in host response or alters host tissue sites as to make them more suitable for pneumococcal adherence and colonisation. We are currently investigating these possibilities.

Our results also indicate that of lack of SP-D contributes to the early onset and increased levels of bacteraemia during pneumococcal pneumonia. It is important to note that SP-D+/+ mice cleared bacteria from their blood by 48 hrs post infection and that the numbers of pneumococci in the blood of both strains of mice reflected their levels in the lung. These results strongly suggest that lung surfactant protein D plays an important role in delaying the appearance of pneumococci in the blood and in limiting their numbers in the bloodstream.

SP-D binds and agglutinates *S. pneumoniae *in the presence of calcium and is thought to enhance mucociliary and phagocytic clearance [11,17]. In addition, binding of SP-D to lipoteichoic acid and peptidoglycan [22] may suggest a role for SP-D in the prevention of bacterial colonisation of the alveolar epithelium. Elimination of these SP-D functions could explain the colonisation of the trachea and nasopharynx, the decreased pneumococcal clearance from lungs and the early onset of pneumococcal bacteraemia observed in SP-D deficient mice in our study.

As reported for other strains of mice [19,23,24], pneumococcal infection was coupled with an influx of neutrophils into the lung tissue of both SP-D+/+ and SP-D-/- mice. This is consistent with the data of LeVine and colleagues [14,15] who also showed that neutrophil accumulation was similar in the lungs of SP-D-/- and SP-D+/+ mice after *H. influenzae *and group B streptococcal infection. In our study, the recruitment of neutrophils in the first 24 hrs post-infection was not affected by the absence of SP-D. However, our results also indicate that the neutrophil response in SP-D deficient mice was not maintained for as long as in wild-type mice. SP-D has been reported as a chemotactic factor for neutrophils *in vitro *[25], and although our data demonstrates that the lack of SP-D does not effect early neutrophil infiltration into lungs, it does clearly affect the longer-term influx of neutrophils as demonstrated by the significant drop in neutrophil infiltration by 48 hrs in SP-D-/- mice. This is not a simple reflection of lung pneumococcal numbers either, as by 24 hrs although there is a significant difference in bacterial CFUs in mice (see figure-3, SP-D+/+ compared to SP-D-/-), the neutrophil numbers in these mice at 24 hrs is not significantly different. Although a similar accumulation of neutrophils was observed in the lungs of both SP-D+/+ and SP-D-/- mice by 24 h after infection, there were significantly greater numbers of pneumococci in the lungs of SP-D-/- mice at this timepoint. This could have resulted in decreased levels of phagocytosis due to the deficiency in the binding and opsonisation of the pneumococcus due to the lack of SP-D, but also could be due to other factors affecting neutrophil activity. For example, as others and we have previously shown, SP-D deficient mice, despite their healthy appearance, develop progressive alveolar proteinosis and have increased numbers of foamy alveolar macrophages [5,18,26]. Thus, it is possible that the excess lipid in SP-D-/- lungs may inhibit the neutrophil respiratory burst, as previously demonstrated *in vitro *[27].

Together with others we have also previously shown that SP-D deficient mice have a 5- to 10-fold increase in the number of apoptotic and necrotic alveolar macrophages compared to wild-type mice, suggesting a contribution of SP-D to immune homeostasis by recognising and promoting removal of apoptotic cells *in vivo *[28,29]. It will be of value to assess the clearance of infected apoptotic neutrophils during pneumococcal infection in SP-D deficient and sufficient mice. We are currently in the process of examining this.

Previous studies have also reported that SP-D inhibits T lymphocyte proliferation and local T cell responses *in vitro *[30,31]. It is therefore noteworthy that we found a heavy infiltration of T lymphocytes in the vicinity of inflamed bronchioles in SP-D deficient lungs at 24 hrs post pneumococcal infection, in contrast to infected SP-D+/+ mice, which exhibited minimal numbers of T cell infiltration. Thus, it appears that SP-D influences T cell infiltration patterns in lungs during pneumococcal infection. It is unclear however, whether SP-D influences T lymphocyte recruitment directly or whether the enhanced T cell infiltration is a consequence of the stimulus of bacteria persisting longer in the respiratory tract of the SP-D deficient mouse. Our previous studies would indicate however, that T cell infiltration is not directly dependant upon pneumococcal numbers as similar colony forming units of pneumococci in lungs and blood of mice can result in totally different T cell infiltration patterns [32]. In addition, in this study we have shown that significantly different pneumococcal numbers can result in significantly different leukocyte infiltration patterns and vice versa.

It has been suggested that SP-D might provide an important link between innate and adaptive immunity, by modulation of antigen presenting cells and T cell function [33] whereby SP-D would enhance the uptake of respiratory pathogens in the alveolar space by recruited antigen presenting cells, whilst suppressing T cell activation in the alveolar space in order to prevent an inflammatory cascade that could damage the local lung airspaces and impair gas exchange [4,33]. Our findings also support an important anti-inflammatory role for SP-D in pneumoccocal infection *in vivo*. Indeed, previous studies have also shown increased pulmonary inflammation, cellular recruitment, oxidant production and decreased macrophage phagocytosis in SP-D deficient mice infected with *Haemophilus influenzae *and group B streptococcus. No decrease in bacterial killing in the lungs of these mice were observed in this study [14], suggesting that other aspects of immunity compensated for the lack of SP-D and cleared the infection effectively. However, after intranasal infection with influenza A virus, SP-D deficient mice showed decreased viral clearance and uptake by alveolar macrophages and increased production of inflammatory cytokines in response to viral challenge [15]. Additional studies are clearly required to further elucidate the role of SP-D in regulating adaptive immune responses *in vivo*.

The potential of truncated recombinant forms of SP-D as a new therapy for infectious and inflammatory diseases has recently been investigated [[34]-35]. Treatment by intranasal administration of SP-D and a 60-KDa recombinant fragment of human SP-D (rSP-D) had a protective effect in a murine model of fungal infection and allergy caused by *Aspergillus fumigatus *[16]. The survival rate of mice increased to 60 and 80% after treatment with SP-D and rSP-D, respectively [16]. In addition, intrapulmonary administration of rSP-D reduced the number of apoptotic and necrotic alveolar macrophages and partially corrected lipid accumulation in SP-D-/- mice [28]. Thus, it would be of a great interest to investigate whether the co-administration of SP-D or truncated rSP-D with *S. pneumoniae *would correct the defects observed in SP-D deficient mice during pneumococcal bronchopneumonia. Administration of SP-D at intervals after infection may also indicate at what stages in the disease process, the protein is most heavily involved. We are currently in the process of investigating these questions.

In summary, the absence of lung surfactant protein D increases the persistence of pneumococcal colonisation and infection in the upper and lower respiratory tract, as well as leading to earlier onset and increased levels of bacteraemia. In addition, the pattern of cellular infiltration into the lungs of SP-D-/- mice following pneumococcal infection is different from SP-D+/+ mice, as characterised by shorter-term neutrophil influx and increased levels of T cell infiltration. SP-D clearly has an important function in the early stages of infection as part of the host immune response to pneumococcal invasion and warrants further study.
